# The Relationship between Gestational Diabetes Metabolic Control and Fetal Autonomic Regulation, Movement and Birth Weight

**DOI:** 10.3390/jcm10153378

**Published:** 2021-07-30

**Authors:** Janine Zöllkau, Laura Swiderski, Alexander Schmidt, Friederike Weschenfelder, Tanja Groten, Dirk Hoyer, Uwe Schneider

**Affiliations:** 1 Department of Obstetrics, Jena University Hospital, Friedrich Schiller University, 07747 Jena, Germany; l.swiderski@gmx.de (L.S.); Friederike.Weschenfelder@med.uni-jena.de (F.W.); tanja.groten@med.uni-jena.de (T.G.); schneider@femme-frauengesundheit.de (U.S.); 2 Biomagnetic Center, Hans Berger Department of Neurology, Jena University Hospital, Friedrich Schiller University, 07747 Jena, Germany; Alexander.Schmidt2@med.uni-jena.de (A.S.); DIRK.HOYER@med.uni-jena.de (D.H.)

**Keywords:** fetal magnetocardiography, fetal autonomic control, heart-rate-variability, perinatal programming, gestational diabetes, metabolic control, birth weight, fetal movement activity

## Abstract

(1) Background: Maternal metabolic control in gestational diabetes is suggested to influence fetal autonomic control and movement activity, which may have fetal outcome implications. We aimed to analyze the relationship between maternal metabolic control, fetal autonomic heart rate regulation, activity and birth weight. (2) Methods: Prospective noninterventional longitudinal cohort monitoring study accompanying 19 patients with specialist clinical care for gestational diabetes. Monthly fetal magnetocardiography with electro-physiologically-based beat-to-beat heart rate recording for analysis of heart rate variability (HRV) and the ‘fetal movement index’ (FMI) was performed. Data were compared to 167 healthy pregnant women retrieved from our pre-existing study database. (3) Results: Fetal vagal tone was increased with gestational diabetes compared to controls, whereas sympathetic tone and FMI did not differ. Within the diabetic population, sympathetic activation was associated with higher maternal blood-glucose levels. Maternal blood-glucose levels correlated positively with birth weight z scores. FMI showed no correlation with birth weight but attenuated the positive correlation between maternal blood-glucose levels and birth weight. (4) Conclusion: Fetal autonomic control is altered by gestational diabetes and maternal blood-glucose level, even if metabolic adjustment and outcome is comparable to healthy controls.

## 1. Introduction

Gestational diabetes mellitus (GDM), defined as any glucose tolerance disorder diagnosed during pregnancy for the first time, affects more than five percent (5.38%) of pregnant women in Germany, with a steep increase being observed during the past 15 years [1]. The metabolic disturbance is caused by peripheral maternal insulin resistance resulting in increased blood glucose levels. Being transferred diaplacentally to the fetus, glucose triggers the fetal pancreas to enhance insulin secretion. In consequence, hyperinsulinemia may lead to growth stimulating effects resulting in macrosomic fetal development [2]. Both perinatal complications and long term metabolic and cardiovascular risk are increased in the offspring of diabetic mothers [3,4].

The metabolic challenge in maternal GDM is reported to affect neurobehavioral aspects in the fetus. Zisser et al. observed differences during movement counts and associated low activity in the fetuses to a tendency for macrosomic development [5]. This so-called fidgety hypothesis was not proven in further studies or using alternative technology. Fehlert et al. performed an fMCG study during a 75 g-oral glucose challenge at 27 weeks of gestation. When retrospectively dividing their collective in women with GDM and no-GDM, they observed differences in the fetal autonomic response to maternal glucose load between both groups [6].

Intrauterine neurobehavioral and autonomic development is characterized by the incremental synchronization of body movements, eye movements and heart rate patterns, particularly during the late second and the third trimesters of gestation [7,8]. A reactive heart rate pattern signaling fetal well-being, classified in the clinical routine as normal according to FIGO (International Federation of Gynecology and Obstetrics), characteristically determines the state of active fetal sleep (synonymously referred to as 2F state [9]). These heart rate patterns are typically accompanied by continuous eye-movements and frequent gross body movements [7,10].

Heart rate pattern underlies the regulation of the autonomic nervous system and hence fetal heart rate patterns are a window into autonomic nervous development in utero. We showed that heart rate variability characteristics and, therefore, developmental rates of fetal autonomic maturation in healthy pregnancies reveal dynamic changes, reaching a developmental milestone at a gestational age of 32 weeks [8,11].

Fetal magnetocardiography (fMCG) delivers a biological signal of high temporal and spatial resolution resembling the typical features of an ECG. Based on fMCG, the fetal heart rate can be determined on a beat-to-beat (RR) base, which is not accessible by standard clinical means [12]. Heart rate variability (HRV) analysis is considered the gold standard to assess autonomic integrity [7,8]. HRV parameters resulting from statistical analysis of beat-to-beat heart rate interval changes, as provided by fMCG, can distinguish branches of the autonomic nervous system (Table 1).

Movements of the fetal heart in relation to the fMCG sensor array result in spatial changes of the recorded cardiac signals and can be used to determine gross fetal body movements [10,16,17,18]. These changes result in amplitude variations and morphological changes of the QRS complex pattern in the multi-channel MCG presentation of the heartbeats induced by the change in position and orientation of the fetal heart. Therefore, fMCG allows the exact and synchronous acquisition of electrophysiological fetal heart activity, leading to indices of vagal and sympathetic HRV and fetal body movements, as well as the classification of the underlying fetal behavioral state.

The present work aims at the overarching analysis of the connection between maternal metabolic, fetal autonomic, movement activity and birth weight during the fetal active state.

## 2. Materials and Methods

Nineteen women pregnant with singletons newly diagnosed with GDM by a standard 75 g oral glucose challenge at 27 weeks of gestation consented to participate in a fMCG follow up and to accompany specialist care in our GDM clinic. Counselling on the triad of blood glucose self-monitoring (Aviva, Accu Chek, Germany), dietary adjustments and the increase of personal activity formed the primary clinical intervention according to German S3 guidelines published in 2018 [19]. Patients were usually seen every 2–4 weeks. The decision on additional insulin treatment was based both on blood-glucose protocols and growth dynamics of the fetus monitored by serial biometric ultrasound scans. Additional insulin treatment was necessary in 10 of the 19 subjects during the later courses of their pregnancies. Monthly follow up by fMCG was aimed at 27, 31, 35 and 39 weeks of gestation. Dropouts led to an average number of 2.4 study monitoring sessions per subject (45 fMCG recordings). Clinical data recorded were maternal weight, height and body mass index at pregnancy entry, weight gain during pregnancy, mean maternal blood-glucose (mMBG) and mean 1 h postprandial maternal blood-glucose (mMBGpp) during the seven days prior to the respective fMCG recording session, HbA1c at term and perinatal outcome data (neonatal sex, birth weight, length, head circumference, mode of delivery, APGAR score at minute five, umbilical artery pH, admission to NICU). Percentiles of neonatal outcome parameters and Z scores to account for gestational age at birth were calculated based on the INTERGROWTH-21 charts [20].

The standard procedure for fetal magnetocardiography has been established at the Biomagnetic Center of the University Hospital of Jena since 2004 [8,11]. A 168-multichannel-magnetometer covering an area of 230 mm in diameter (Argos 200, ATB, Chieti, Italy) based in a magnetically shielded room is positioned above the maternal abdomen without contact after sonographic localization of the fetal heart. The recording session is standardized to 30 min. Diurnal variations during daytime have no impact on HRV results as long as an adequate assessment of the neurobehavioral state is performed; therefore, recording sessions were scheduled between 12 am and 3 pm in the early afternoon [21].

Recorded heart rate traces were assessed for the number of distinct five-minute intervals of a reactive heart rate pattern indicating active sleep (2F state). HRV analysis and determination of the fetal movement index (fMI) were calculated accordingly [13]. The considered parameters and their meaning are listed in Table 1.

Work steps to calculate fetal HRV parameters and to identify fetal movements included an independent component analyses with an automatic recognition of fetal components in order to reconstruct the fetal MCG (fMCG), and rate of change of the Hilbert Amplitude algorithm for the identification of the fetal heart beat positions and the resulting RR intervals [22].

**Figure 1:**
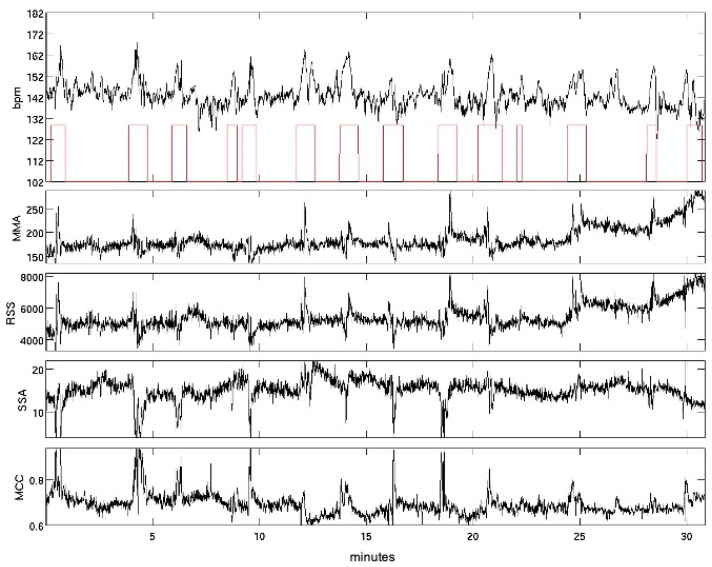
Calculation of fetal movements from MCG recording. Heart rate trace (black line) and sections of identified body movements (red boxes). Four calculation approaches were combined to detect spatial shift of the cardiac vector within the sensor array in all dimensions: Minimum Maximum Amplitude (MMA), L2-Norm of the heart vectors (RSS), Signal Space Angle (SSA) and Moving Correlation Coefficient (MCC) (details in [17,22]).

Based on the fMCG and the heartbeat positions, four movement graphs were calculated, quantified and combined in to one fetal movement index (fMI). The fMI is defined as the percentage of time that the fetus is moving. Two of the used graphs Minimum Maximum Amplitude and L2-Norm of the heart vectors primarily quantify amplitude changes, whereas Signal Space Angle and Moving Correlation Coefficient tend to identify changes of the ORS complex pattern. These four graphs quantify most of the fetal body movement types. Figure 1 illustrates the fMI, and calculation details can be found in [17].

Study group results were compared to a collective of normal singleton pregnancies according to standard maternity care in Germany, restricted to a gestational time span from 27 to 39 weeks of gestation and excluding women with GDM. The respective 167 cases were selected from the Jena fMCG database (537 recordings, average 3.2 recordings per subject) [8,11]. All analyzed heart rate traces of the study and control groups were clinically unremarkable and diagnosed to be “normal” according to FIGO.

Statistical calculations were carried out using SPSS 25 (IBM, SPSS Statistics, Armok, New York, United States of America). Statistical significance was assumed at a *p* < 0.05 and statistical trends at a *p* < 0.1. T-tests or Mann-Whitney-U-tests were applied as appropriate for the resulting data types. Normal distribution was evaluated using the Kolmogorov-Smirnoff-Test. Fetal HRV parameters were analyzed irrespective of gestational age and subdivided into early (before 32 weeks of gestation) and late recordings (after 32 weeks of gestation). General estimating equations (GEE) were used to consider intraindividual dependencies. The patient’s ID was used as a subject variable. To involve the development of the fetal autonomic control and the varying timespans between the fMCG recordings of one patient, gestational age at the timepoint of recording was included as a within-subject variable. Influencing factors were included step by step into these models. At first the influence of movement (fMI) was the sole factor, second the influence of blood-glucose (mMBG or mMBGpp were separately considered) was added, and in the third step an interaction term of movement and glucose level (fMI*mMBG; fMI*mMBGpp) was added to the analyses. To compare the regression coefficients of Beta (RcB) within the models, the investigated parameters were Z-normalized.

The study was approved by the Ethics committee of the Friedrich-Schiller-University of Jena (1104-04/03).

## 3. Results

Clinical data of the 19 pregnant women with GDM (study cohort) and the 167 pregnant controls accompanying longitudinal fMCG monitoring are displayed in Table 2. While maternal weight and body-mass indices were higher in GDM patients, no other differences in maternal characteristics or neonatal outcome were observed.

**Figure 2:**
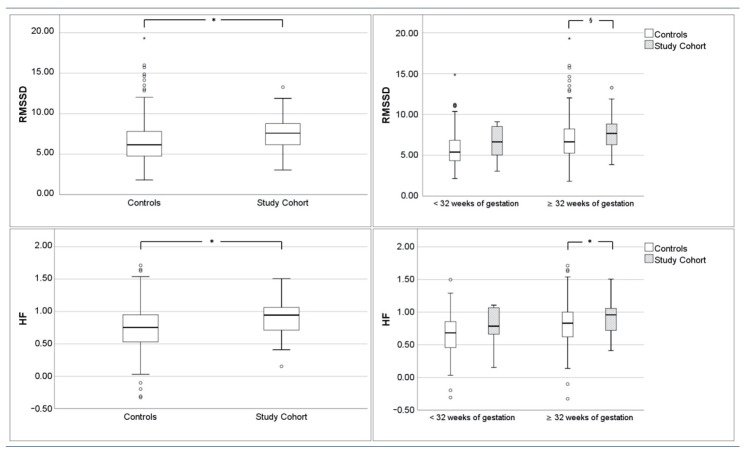
Boxplots of RMSSD and HF representing vagal tone over the entire gestational period (left panels) and <32/≥32 weeks of gestation (right panels) comparing study and control cohorts, (*p* < 0.05 marked with *; trend *p* < 0.1 marked with §; *t*-test).

The results of HRV and fMI analyses, irrespective of gestational age and subdivided for early and late pregnancy, are listed in Table 3. Notably, parameters representing vagal modulation (RMSSD, HF) were increased in the study cohort. The effect was pronounced beyond 32 weeks of gestation (Figure 2).

The effects of fetal activity and blood-glucose control on sympathetically interpreted fetal HRV in the study cohort are summarized in Table 4. For vagally interpreted HRV no significant results were found. There was a positive correlation between fMI and parameters representing mainly sympathetic activity (see also Table 4 for selected details; fMI-VLF in the study cohortRcB = 0.205, *p* < 0.01). In addition, both interaction terms fMI*mMBG and fMI*mMBGpp influenced the parameters of sympathetic activity.

**Figure 3:**
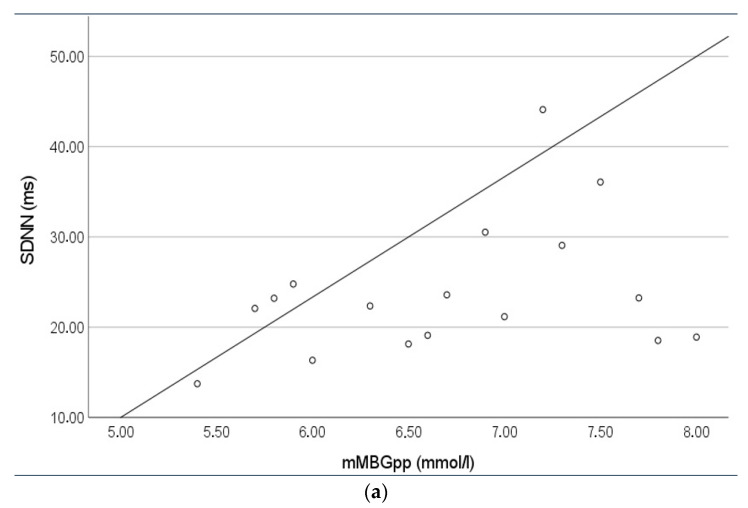

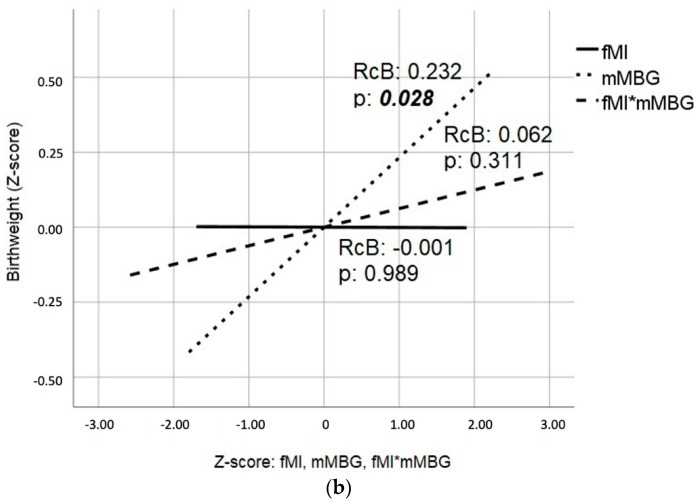
(**a**) Regression analysis between mean one-hour postprandial maternal blood-glucose (mMBGpp) levels of the seven days prior to the respective fMCG recording and SDNN as a marker of overall fetal HRV representing mainly sympathetic activity (RcB 0.339; *p* = 0.009). (**b**) Z-score normalized values for birthweight, fetal movement index (fMI), mean maternal blood-glucose (mMBG) of the seven days prior to the respective fMCG recording and the interaction term fMI*mMBG (general estimating equation model). Birthweight was positively related to mMBG (RcB 0.232; *p* = 0.028) but not fMI alone (RcB −0.001; *p* = 0.989). The interaction fMI*mMBG attenuated the effect between mMBG and birthweight (RcB 0.062; *p* = 0.311).

The same associations could be reproduced in the control cohort (data not shown in detail). While mean 1 h postprandial maternal blood-glucose levels were positively associated with the sympathetic branch (Figure 3a), we did not observe such a relation for mean maternal blood-glucose levels.

In the study cohort, birth weight and mean maternal blood-glucose levels during the week prior to fMCG recording (both overall and 1 h-post prandial) were positively correlated with birth weight (mMBG: RcB = 0.232, *p* = 0.028; mMBGpp: RcB = 0.080, *p* = 0.025). In contrast, fMI and birthweight (RcB = 0.002, *p* = 0.962) were not correlated. Figure 3b illustrates the influences on birth weight. Interestingly, the interaction between fMI and mean maternal blood-glucose seemed to attenuate the effect glucose levels had on birth weight in our sample (fMI*mMBG: RcB = 0.062, *p* = 0.311, fMI*mMBGpp: RcB = 0.02, *p* = 0.422).

In the control group, an exceedingly small positive correlation between fMI and birth weight was found (RcB = 0.062, *p* = 0.035; data not shown in detail).

## 4. Discussion

In our study (that to our knowledge is the first to perform longitudinal monitoring of fetal HRV in a cohort of patients with GDM) we found several deviations in autonomic regulation in comparison to a large cohort representing the physiological variation of a normal population. Maternal GDM was associated with an increase of vagal modulation of the heart rate patterns during active sleep (2F state) beyond 32 weeks of gestation. The observations were confined to a three-hour postprandial window in the early afternoon. However, higher average maternal glucose levels seemed to enhance sympathetic activity. All heart rate traces of both the study and control cohorts were unremarkable in clinical terms. As proofs of our concepts, (a) maternal glucose levels and higher birth weights were correlated and (b) activity of the sympathetic branch of the fetal autonomic nervous system went along with increased fetal activity.

The association between the values of fetal activity and birth weight appears to be low, or nonexistent, as a trend, thus disproving the fidgety hypothesis. Based on the observed reversal of the positive correlation between long-term glucose levels and birth weight by higher rates of fetal movement activity, one may hypothesize that fetal movements attenuate weight gain in relation to maternal metabolic control.

Our results, hinting a higher fetal vagal tone in the study cohort, are somewhat in contrast to the findings presented by Fehlert et al. [6]. In their study, an increase of sympathetically driven fetal HRV was found at the one-hour interval under metabolic challenge during a standard 75 g oral glucose test that overshot into reverse at two-hours in those subjects who were diagnosed to have GDM with the same test [6]. Reduced fetal capacity to compensate metabolic stress has been discussed to explain these results. Our finding of higher sympathetic activity in association with higher maternal long-term blood-glucose monitoring results point towards the same direction. The difference in our study is that monitoring occurred during a certain time window in the early afternoon but with no strict relation to a defined metabolic challenge; a situation that more closely resembles routine clinical management settings in later pregnancy. fMCG monitoring was scheduled after a gestational diabetes care appointment. Our finding of an increased vagal tone is backed by the observation of increased fetal thoracic movements during the postprandial periods. Thoracic excursions go along with respiratory sinus arrhythmia, particularly displayed in RMSSD and HF [23]. Fetal insulin levels are higher in GDM. In adults, nasal insulin application led to higher vagal activities in the brain. Insulin is released physiologically especially after meals. The digestive process is modulated mainly vagally [24]. Thus, it is conceivable that fetuses in GDM may respond to higher maternal blood-glucose levels with increased insulin secretions and, therefore, show higher vagal tone. Mean maternal blood-glucose levels were used to describe the therapeutic adjustment during the week prior to the fMCG session. To investigate the direct interaction between maternal blood-glucose and the fetal ANS a simultaneous survey is advisable.

Maternal weight and body mass indices were significantly higher in our study cohort compared to a collective of normal controls, which could be expected since obesity is one of the major risk factors for developing GDM [1]. In fact, in previous studies we observed a higher impact of maternal body mass indices and weight gain during pregnancy on maternal and perinatal outcomes, irrespective of the diagnosis of GDM, than by the diagnosis of GDM alone [25,26]. On the contrary, neonatal outcome was favorable in our cohort, and did not observe significant differences in comparison to controls. Up to 15–45% of the offspring of diabetic mothers are reportedly at risk to be large for gestational age, and between two and eight percent may be burdened with respiratory or metabolic distress post natum [27,28]. We may, therefore, conclude that intensive antenatal monitoring of blood-glucose levels in combination with counselling on dietary recommendations, as well as initiation of insulin treatment in about 50% of cases, seems to have reduced the likelihood of diabetic complications in this collective. Patients, though, were not recruited on clinical grounds but on their preparedness to consent to additional, purely scientific monitoring. Therefore, the sample is not representative, and positive selection bias regarding compliance and adherence to clinical advisement is likely.

Fetal movements as an expression of activity in combination with distinct heart rate patterns are the cornerstones to assess the fetal neurobehavioral state of activity [10]. The state of these patterns is one of the fundamental markers indicating normal functional maturation [7]. The more active the fetus is, the higher the sympathetic tone [29].

Maternal blood-glucose levels were strongly associated with birth weight, as expected. Interestingly, when the quantity of fetal movements was normalized as the ‘fetal movement index’ and considered statistically, this association was attenuated. Such a possible compensation effect was postulated by Zisser et al. [5]. This result could be, at least in part, an explanation of why some of the neonates who featured identical metabolic control turned out macrosomic while others did not.

We observed a small but statistical association between fMI and higher birthweight in the normal population that we did not expect. Methodical idiosyncrasies are the most likely explanation: (1) both small-for-gestational age (< than 10th percentile) and fetal growth restriction disqualified inclusion in the control cohort on the data base search, and (2) Z score normalization based on the international Intergrowth-21 charts led to a skewered distribution of birth weights in a German population disproportionate to LGA, as can be seen from Table 3.

The presented study uses fMCG as a highly sophisticated and resource-demanding procedure technically, enables performance of beat-to-beat analysis of the heart rate and detection of movements of the fetal heart relative to the sensor concurrently. The method described here is blind to isolated movements of the limbs. fMCG is far too costly and specialist driven to gain worldwide distribution. Therefore, efforts should be encouraged to assess fetal neurobehavioral development based on routine clinical applications [30].

From a scientific point of view, fetal heart rate monitoring in GDM patients might be warranted with to confirm, extend and generalize the findings from our relatively small cohort of 19 patients.

## 5. Conclusions

This is, to our knowledge, the first investigation into long-term electrophysiological fetal heart rate monitoring of GDM patients. Fetal autonomic control was altered by gestational diabetes (higher fetal vagal tone, pronounced after 32 weeks of gestational age) and maternal blood-glucose level (fetal sympathetic activation), even with comparable metabolic adjustment and outcome to healthy controls. Fetal movements activity by FMI was not correlated with birth weight, but attenuated the positive correlation between maternal blood-glucose levels and birth weight.

Fetal heart rate monitoring offers a noninvasive opportunity to access fetal autonomic control alterations in GDM patients. The value of this technique in context of GDM routine clinical management, as well as possible neurodevelopmental implications, has to be addressed in further examinations.

## Figures and Tables

**Table 1:** Selected parameters of beat-to-beat heart rate variability [11,13,14,15].

Parameter	Calculation	Interpretation
Parameters of the Time Domain		
SDNN (ms)	Standard deviation of NN intervals	Overall (sympathetic and parasympathetic influences), predominantly sympathetic
RMSSD (ms)	Root mean square of successive NN-interval differences	predominantly vagal activity
Actamp20 (bmp)	20–95 inter-percentile distance of the trend-corrected NN interval series in beats per minute	Overall (sympathetic and parasympathetic influences), predominantly sympathetic
pNN5 (%)	Percentage of successive NN-interval differences that are > 5 ms	Predominantly vagal activity
Parameters of the Frequency Domain		
VLF (ms^2^)	Very low frequency, spectral power of the frequency band from 0.02–0.08 Hz	Baseline-fluctuations, sympathetic influence
HF (ms^2^)	High frequency, spectral power of the frequency band from 0.4–1.7 Hz	vagal influences, respiratory sinus arrhythmia

**Table 2:** Summary of the accompanying data of the study cohort (GDM) and comparison to the controls.

	Study Cohort (GDM)			Controls			
	No.	mean/%	SD	No.	mean/%	SD	*p*
**Maternal Characteristics**							
Maternal age (years)	17	30.1	4.7	147	28.8	4.7	0.92
Maternal weight (kg)	16	80.6	14.4	146	67.1	11.7	<0.01
Height (cm)	16	167.1	6.0	146	167.1	6.7	0.84
BMI (kg/m^2^)	16	28.9	4.8	146	24.0	3.8	<0.01
Pregnancy weight gain (kg)	9	12.33	7.25				
mMBG (mmol/L)	16	5.99	0.51				
min/max MBG (mmol/L)	16	5.1/7.1					
mMBGpp (mmol/L)	12	6.62	0.65				
min/max mMBGpp (mmol/L)	12	5.4/8.0					
Hb1Ac (%)	10	5.31	0.29				
min/max HbA1c (%)	10	4.8/5.8					
**Neonatal Outcome**							
birthweight (g)	17	3353.5	509.5	147	3402	539.9	0.84
Z score birthweight	17	0.45	0.99	147	0.40	0.94	0.73
percentile birthweight	17	60.2	26.5	147	61.4	27.1	0.83
LGA > 90th Percentile LGA > 4000 g	4 1	23.5% 5.9%		25 17	17% 11.6%	2.9	0.06
head circumference (cm)	17	34.2	1.9	147	34.4	1.8	0.84
APGAR 5	17	9.1	0.6	147	9.1	0.8	0.40
pH umbilical artery	17	7.27	0.25	147	7.25	0.07	0.80
*Newborn sex*	17			152			
male	9	52.9%		68	44.7%		0.61 *
female	8	47.1%		84	55.3%		
**Mode of Delivery**							
spontaneous vaginal	10	58.8%		106	69.7%		0.41 *
caesarean section	6	35.3%		34	22.4%		
operative vaginal	1	5.9%		12	7.9%		
admission to NICU	4	23.5%		24	16.3%		0.49 *

There are differences with respect to maternal weight and body mass indices. (No.—number of cases with available data; mean—mean value; SD—standard deviation; BMI—body mass index, mMBG—mean maternal blood glucose, mMBGpp—mean 1 h postprandial maternal blood glucose, LGA—large for gestational age); *p*-values were calculated using Mann-Whitney-U- or *t*-test depending on normal distribution, * *p*-values were calculated using Fisher-Yates test.

**Table 3:** Results of HRV analysis and fMI (± standard deviation) in the study cohort (GDM) and comparison to controls. Overall (independent of gestational age), <32 weeks of gestation and ≥32 weeks of gestation, *p*-values were calculated using Mann-Whitney U or *t*-test depending on normal distribution.

	Overall			<32 Weeks of Gestation			≥32 Weeks of Gestation		
	GDM	Controls	*p*	GDM	Controls	*p*	GDM	Controls	*p*
**S** **ympathetic**									
SDNN (ms)	22.9 ± 6.5	22.1 ± 7.3	0.39	22.1 ± 6.8	19.5 ± 6.5	0.19	23.2 ± 6.4	24.0 ± 7.2	0.47
Actamp20 (bpm)	18.1 ± 5.7	17.8 ± 5.8	0.81	17.0 ± 5.4	15.1 ± 5.0	0.25	18.4 ± 5.8	19.7 ± 5.6	0.23
VLF (ms^2^)	2.2 ± 0.2	2.1 ± 0.3	0.38	2.1 ± 0.3	2.0 ± 0.3	0.17	2.2 ± 0.2	2.2 ± 0.3	0.54
**Vagal**									
RMSSD (ms)	7.4 ± 2.1	6.6 ± 2.4	<0.01	6.6 ± 2.1	5.8 ± 2.0	0.16	7.7 ± 2.1	7.1 ± 2.6	0.06
pNN5 (%)	0.30 ± 0.10	0.28 ± 0.12	0.13	0.24 ± 0.11	0.22 ± 0.1	0.51	0.36 ± 0.10	0.36 ± 0.12	0.98
HF (ms^2^)	0.9 ± 0.3	0.8 ± 0.3	<0.01	0.8 ± 0.3	0.7 ± 0.3	0.18	1.0 ± 0.3	0.8 ± 0.3	0.04
**Movement**									
fMI (%)	36.5 ± 20.4	39.9 ± 21.7	0.34	31.3 ± 21.5	35.0 ± 20.8	0.49	38.0 ± 20.1	43.3 ± 21.7	0.23

**Table 4:** Results of the general estimating equation (GEE) analysis regarding the associations between fetal movement index (fMI), mean maternal blood-glucose (mMBG), mean one-hour post prandial maternal blood-glucose levels (mMBGpp) during the seven days prior to fMCG recording, and the respective interaction terms fMI*mMBG and fMI*mMBGpp to the parameters SDNN and Actamp20 in the study cohort: regression coefficients of Beta (RcB).

	SDNN		Actamp20	
	RcB	*p*	RcB	*p*
fMI	0.490	<0.001	0.553	<0.001
mMBG	0.185	0.121	0.112	0.359
mMBGpp	0.339	0.009	0.282	0.016
fMI*mMBG	0.231	0.026	0.272	0.035
fMI*mMBGpp	0.302	0.025	0.264	0.138

## Data Availability

The data presented in this study are available on request from the corresponding author. The data are not publicly available due to patient privacy reasons.
